# Targeting Cyclic AMP Signalling in Hepatocellular Carcinoma

**DOI:** 10.3390/cells8121511

**Published:** 2019-11-25

**Authors:** Mara Massimi, Federica Ragusa, Silvia Cardarelli, Mauro Giorgi

**Affiliations:** 1 Department of Life, Health and Environmental Sciences, University of L’Aquila, 67100 L’Aquila, Italy; fragusa@unite.it; 2 Department of Biology and Biotechnology “Charles Darwin”, Sapienza University of Rome, 00185 Rome, Italy; silvia.cardarelli@uniroma1.it

**Keywords:** phosphodiesterase, cyclase, PDE, PKA, EPAC, hepatocyte, GPCR, HCC, PDE inhibitors, cancer

## Abstract

Hepatocellular carcinoma (HCC) is a major healthcare problem worldwide, representing one of the leading causes of cancer mortality. Since there are currently no predictive biomarkers for early stage diagnosis, HCC is detected only in advanced stages and most patients die within one year, as radical tumour resection is generally performed late during the disease. The development of alternative therapeutic approaches to HCC remains one of the most challenging areas of cancer. This review focuses on the relevance of cAMP signalling in the development of hepatocellular carcinoma and identifies the modulation of this second messenger as a new strategy for the control of tumour growth. In addition, because the cAMP pathway is controlled by phosphodiesterases (PDEs), targeting these enzymes using PDE inhibitors is becoming an attractive and promising tool for the control of HCC. Among them, based on current preclinical and clinical findings, PDE4-specific inhibitors remarkably demonstrate therapeutic potential in the management of cancer outcomes, especially as adjuvants to standard therapies. However, more preclinical studies are warranted to ascertain their efficacy during the different stages of hepatocyte transformation and in the treatment of established HCC.

## 1. Introduction

Hepatocellular carcinoma (HCC) is one of the most common tumours of the liver and a leading cause of cancer-related mortality in the world [1]. Owing to a deficiency of biomarkers for early diagnosis, tumour resection is very often performed late during the disease process, with radiotherapy and chemotherapy remaining the only options.

The most widely used biomarker for HCC is serum α-fetoprotein (AFP), but its sensitivity remains below 60% [2]. Chemotherapy treatments include the use of drugs that target kinases and growth factor receptors. Sorafenib is currently considered the most effective drug for unresectable HCC. Besides being effective against VEGF and PDGF tyrosine kinase receptors, sorafenib acts downstream on the Ras/Raf kinases, thus regulating cellular proliferation and angiogenesis [3]. Nevertheless, the effectiveness of sorafenib and other available chemotherapics allow for only partial control of the disease, also due to the development of drug resistance. The search for new treatments or adjuvants able to improve the chance of survival of patients affected remains of utmost importance [4,5].

HCC is a heterogeneous disease with a very complicated aetiology. Important factors that predispose to HCC are viral hepatitis B (HBV) and/or C (HCV) infections, alcohol consumption, aflatoxin, obesity, non-alcoholic fatty liver disease and, especially, liver cirrhosis [6]. A chronic hepatic injury commonly develops to fibrosis, due to accumulation of extracellular matrix (ECM), consequent hypoxia of the tissue and induction of HIF-1α (hypoxia-inducible factor-1α). The fibrotic condition, along with the activity of cells in the surrounding microenvironment (i.e., endothelial cells, immune cells and fibroblasts), are crucial factors in the development of HCC [7]. In addition to extrinsic factors, epigenetic alterations and genetic mutations, inherited or acquired, also contribute to the disease [6,8]. For instance, the gene promoter of telomerase reverse transcriptase (TERT) and the p53 gene are mutated in most HCCs. Mutations are also found in the multidrug resistance (MDR1) and P-glycoprotein gene products [9]. Furthermore, elevated expression of non-coding RNAs, such as long non-coding RNA (lncRNA), micro RNA (miRNA) and circular RNA (circRNA) have also been found to promote HCC progression [8]. 

As with other tumours, several signalling pathways are involved in the neoplastic transformation process, which eventually give rise to tumour development and metastasis formation. These pathways include IGF, Hedgehog, Wnt/β-catenin, PI3 K/AKT/mTOR and RAS/RAF/MAPK, as well as VEGFR and EGFR/RAS/MAPK pathways [3]. Overexpression of PI3K, RAS, EGFR and MAPK proteins is also found in fibrolamellar HCC (FL-HCC), a primary liver cancer that arises in young people without a causal liver disease, distinct from the classic, adult HCC [10]. For instance, alpha-fetoprotein is not elevated in most cases of FL-HCC, which also show normal levels of p53 and β-catenin. A unique feature of FL-HCC is instead the production of a chimeric enzyme able to recruit heat shock protein 70 (Hsp70), thus triggering oncogenic signalling. Chimeric DNAJ-PKAc consists of a chaperonin-binding domain (DNAJ) fused to the Cα subunit of protein kinase A (PKA), an important effector of cAMP signalling [11].

Recently, the modulation of cyclic adenosine monophosphate (cAMP) has been shown to be of great interest in the control of cell proliferation in different cell lines, also thanks to the fact that the intracellular levels of this molecule can be relatively easily controlled pharmacologically. It has become well accepted, in fact, that this second messenger carries out metabolic control as well as direct control of cell proliferation in normal and transformed cells. Specifically, the increase in intracellular cAMP levels is paralleled by an increase in proliferation in some cell types, while in others, including transformed hepatocytes, it generally exerts a negative control, thus resulting in significant down regulation in hepatocarcinoma cells [12,13,14,15].

The cAMP level is spatially and temporally regulated by a balance between cyclase and phosphodiesterase activities. Cyclases are enzymes that synthesize cAMP from intracellular ATP. Phosphodiesterases (PDEs) are enzymes that regulate intracellular levels of cAMP by controlling its rate of degradation. The availability and specificity of natural or synthetic molecules isolated in recent years by many pharmaceutical companies with minimal side effects make PDEs by far the most important targets for the pharmacological control of cAMP and of the numerous intracellular cAMP-dependent effectors. 

Phosphodiesterases are classified in 11 isoform families (PDE1-PDE11). Some control the intracellular levels of both cyclic nucleotides (PDE1, 2, 3, 10, 11), while others are specific for cAMP degradation (PDE4, 7, 8) [16,17].

The purpose of this review is to outline the state of the art of the research that for more than 30 years has been focused on the potential role of cAMP and its dysregulation in malignant hepatocytes, using experimental models treated with receptor agonists involved in this signalling pathway, or with molecules able to interfering with the metabolism of cAMP, or with cAMP analogues capable of determining cAMP pathway activation.

## 2. Physiologic Effectors of cAMP in Hepatocytes

The signalling pathway involving the second messenger cAMP begins with its synthesis by adenylate cyclase (AC), a heterogeneous class of enzymes (AC1–AC10) which, with the sole exception of AC10, are activated by membrane G protein-coupled receptors (GPCR). In contrast, AC10 is localized at the cytosolic level and activated by high levels of bicarbonate and calcium ions [18].

**Figure 1:**
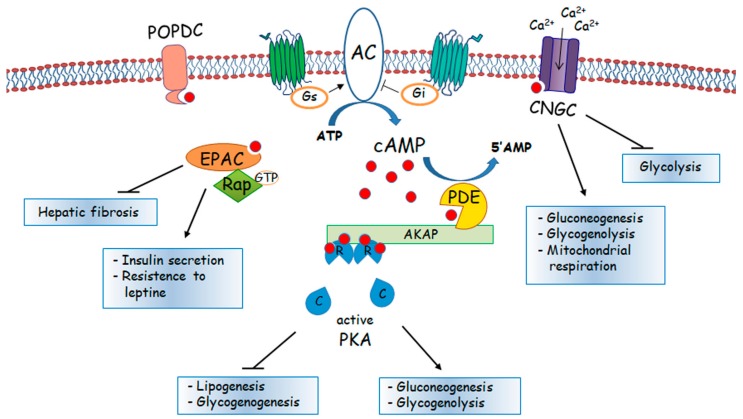
Schematic representation of cAMP formation/degradation and of cAMP target proteins. POPDC, Popeye domain containing protein, AC, adenylyl cyclase; Gs, stimulatory G protein; Gi, inhibitory G protein. CNGC, cyclic nucleotide-gated channels; EPAC, exchange protein directly activated by cAMP; Rap-GTP, GTP-binding Ras related protein; AKAP, A-kinase anchoring proteins; PDE, phosphodiesterase; PKA, cAMP-dependent protein kinase.

In the liver, one of the most well-known metabolic effects involving an increase in cAMP is caused by the pancreatic hormone glucagon, which following its binding to a specific GPCR and the activation of a complex sequence of effectors, leads to the demolition of glycogen in hepatocytes and the release of glucose, which is subsequently introduced into the circulation. The complex signal transduction pathway triggered by glucagon involves activation of protein kinase A (PKA), also known as cAMP-dependent protein kinase. PKA is composed of two catalytic subunits and two regulatory subunits. In its inactive form, the regulatory subunits inhibit the kinase activity of the catalytic subunits (Figure 1).

Spatial and temporal coordination of PKA signalling is achieved by association with scaffolding proteins (AKAPs), which constitute a family of about 20 molecules. Through a specialized domain, AKAPs anchor in different subcellular microdomains at targeting sites, thus assembling macromolecular complexes and recruiting a variety of signalling proteins, including other kinases, PDEs, phosphatases and small G proteins [19]. PKA becomes active in the presence of micromolar concentrations of cAMP, which upon binding to the regulatory subunits induces their dissociation from the catalytic domains. 

The result is the direct activation/inhibition of enzymes involved in glycogen metabolism and gluconeogenesis, such as glycogen phosphorylase, glycogen synthase and fructose 2,6-bisphosphatase [20], and stimulation of the transcription of gluconeogenic enzymes, such as glucose 6-phosphatase (G6Pase), phosphoenolpyruvate carboxykinase (PEPCK) and pyruvate carboxylase (PC) [21].

In hepatocytes, PKA also mediates the inhibition of lipogenesis through the phosphorylation, and consequent inhibition, of key enzymes in fatty acid synthesis, such as acetyl-CoA carboxylase and pyruvate dehydrogenase [22]. Insulin can reverse the phosphorylation levels of these enzymes, stimulating lipogenesis and decreasing glucose production. 

In 1998, the exchange cAMP activated proteins (EPAC) were identified as a new family of molecules able to mediate cAMP signalling, which emerged as new cAMP targets, alternative or concurrently to the classical target PKA [23,24]. 

In the liver, the role of EPAC has only recently been investigated and is still far from becoming completely clear; however, it has been widely demonstrated that the cAMP/EPAC pathway is deeply involved in maintaining body metabolic homeostasis, for example by increasing insulin secretion or by inducing resistance to leptin [25,26,27]. Recent evidence also demonstrates the role of EPAC as a regulator of hepatic fibrosis, by contrasting accumulation of the modified extracellular matrix as well as proliferation and migration of hepatic stellate cells [28,29]. EPAC protein consists of two structural halves connected by a central “switchboard” region. While the N terminal regulatory part of the protein is responsible for the binding to cAMP, the C-terminal part contains the nucleotide exchange factor activity. Low basal levels of cAMP keep EPAC in an auto-inhibitory conformation, with the N-terminal portion folded on top of the C-terminal element and consequently blocking the active site. The binding of cAMP to the EPAC N-terminal end induces conformational changes that make the catalytic site accessible [30,31], also leading to the exposure of a lipid binding module that targets EPAC1 to the plasma membrane [32,33]. In fact, currently two EPAC isoforms are known, EPAC1 and EPAC2; both are cellular sensors of cAMP but with a different pattern of tissue expression. EPAC1 is ubiquitously expressed while EPAC2 is highly expressed in liver, brain, pancreas and adrenal gland. EPAC activation, after cAMP binding, induces a signalling cascade via its downstream effectors, Rap1 and Rap2, members of the Ras superfamily. These GTPase proteins are involved in many physiological functions, such as cellular adhesion and cell-cell junction organization, whose alterations could lead to cellular transformation and metastasis processes. 

Besides PKA and EPAC, also various cyclic nucleotide-gated channels (CNGC) have been identified as intracellular targets that bind cAMP through conserved cAMP-binding domains. These channels are extensively studied in the heart, where CNGCs are involved in heart rate regulation, and in retinal and olfactory tissues, where CNGCs take part in the transduction pathway of the sensorial signal [34,35].

More recently, Popeye domain containing (Popdc) proteins, which bind cAMP with high affinity, have been identified as a new class of cAMP effectors. They are expressed in a variety of tissues and interact with different classes of intracellular proteins in several subcellular compartments. Several studies have demonstrated that Popdc proteins are involved in cell adhesion and motility and are downregulated in several kind of cancers, although their involvement in liver cancer has not been directly investigated [36].

Despite CNGCs being rarely investigated in the liver, biochemical evidence supports the presence of CNGC also in human hepatic cells [37], where they localize in the plasma membrane, as in the rod cells of the retina [38]. These studies strongly define the function of CNGC in the liver; they open in response to an increase in cyclic AMP levels and lead to inward calcium fluxes. An increase in cytosolic Ca^2+^ levels suppresses the glycolytic pathway and stimulates gluconeogenesis, as suggested by Gevers and Krebs [39]. Calcium also stimulates glycogenolysis and mitochondrial respiration, increasing ATP levels, which are important requisites for gluconeogenesis [40,41,42]. It was also demonstrated that inhibition of these ion fluxes blocks the stimulation of gluconeogenesis by cAMP and hormones, such as glucagon, confirming the involvement of CNGC in liver metabolism [43]. 

## 3. Pharmacological Modulators of the cAMP Pathway

Modulation of cAMP signalling by targeting the main effectors along the pathway can contribute to the treatment of numerous human diseases. Cyclic AMP derivates, adenylate cyclase activators, and PKA, EPAC or PDE inhibitors have been largely used in biochemical research of various pathologies. 

The cAMP analogue, dibutyryl cAMP, a synthetic non-hydrolysable compound, membrane permeant, also acts as a modulator of the endogenous concentration of cAMP. Dibutyryl cAMP was introduced in early clinical trials as a strong PKA activator for treatment of congestive heart failure, wounds and inflammation [44,45,46]. 

Forskolin, a plant-derived adenylate cyclase activator, is the most widely used inducer of cAMP formation. It showed therapeutic potential effects in cardiac and liver fibrosis [47,48], in the treatment of glaucoma [49], and also as a dietary supplement for weight loss and obesity reduction [50]. Forskolin has also proven to be a potential drug candidate for cancer therapy, being able to induce growth suppression and apoptosis in several tumorigenic cell types [51]. In this context, forskolin acts as an adenylate cyclase activator and as a potent Hedgehog (Hh) signalling inhibitor. Constitutive activation of the Hh signalling pathway can lead to malignant tumours by a mechanism that remains largely unknown. Forskolin suppresses Hh signalling mostly by inhibiting the expression of Gli1 and Gli2, positive activators of downstream hedgehog target genes. Recent studies have demonstrated that forskolin, by increasing cAMP levels, induces the activation of PKA with a consequent phosphorylation and proteasomal degradation of Gli-1 and Gli-2 [52,53]. Nonetheless, although data already available allow us to predict very promising possibilities in terms of therapeutic development, it appears that compounds targeting adenylate cyclase, or effectors of the synthetic pathway, are still largely undervalued and understudied.

As previously described, cAMP signalling is strongly dependent on both PKA and EPAC activity. H89 is a commonly used PKA inhibitor. Many studies demonstrated the efficacy of H89 in the treatment of inflammation in reducing the immune response [54,55], but also for its anti-tumour effects on various cancer cells [56]. Indeed, the inhibition of PKA through H89 suppressed the increase of the transcription factors FBP1 induced by PGE2. Consequently, the loss of FBP1 function abrogates c-myc expression and arrests the proliferation of liver cancer cells [57]. An EPAC-specific cAMP analogue (8CPT-2Me-cAMP), able to discriminate between EPAC and PKA pathways, has also been developed and widely used in different studies [58,59]. High throughput screening of diverse non-cyclic nucleotide molecule libraries allowed the identification of several EPAC specific inhibitors [60]. The inhibitor ESI-09 has been shown to be effective in suppressing pancreatic cancer metastasis in a mouse model [61].

A different way to affect intracellular cAMP levels is by interfering with the degradative pathway via phosphodiesterase enzymes (PDEs). PDEs are attractive drug targets with significant therapeutic potential [16]. While some isoforms are specific for cAMP degradation (PDE4, 7, 8), most PDEs (PDE1, 2, 3, 10, 11) hydrolyse both cyclic nucleotides, influencing processes dependent on both cAMP and cGMP [17]. 

The hydrolytic activity of this “dual-substrate PDE” is mutually influenced by both substrates, to the point that they can be considered not as simple “poorly specific” degradation enzymes, but rather as active effectors of cAMP, just like EPAC, PKA and CNGC. Specifically, the kinetic and inhibitory parameters of the “dual-substrate” PDE10 suggest a role of cAMP as an effector in regulating the functions of cGMP. For example, the binding of cAMP to the N-terminal domain of PDE10 allosterically inhibits the hydrolytic activity of PDE10 on cGMP [62,63,64].

The PDE4 family that selectively hydrolyses cAMP represents the largest PDE family, encoded by four different genes (PDE4A, 4B, 4C and 4D), which have different promoters and give rise in humans to more than 25 known isoforms through alternative splicing [64,65]. PDE4 enzymes critically control multiple intracellular signalling pathways that can be altered in many pathological conditions, including cancer. PDE4 has therefore been used successfully as a therapeutic target for various inflammatory diseases and for the treatment of depression [66]. Rolipram was one of the first specific molecules to be synthesized for this class of enzymes and has also become the archetype for the synthesis of new and more selective PDE4 inhibitors, such as DC-TA-46 [67,68], roflumilast [69] and GEBR-7b [70]. Therefore, the use of potent and selective inhibitors of PDE4 isoenzymes may be used to reverse upregulation of the PDE and thus to reverse pathology [12,14]. GEBR-7b is of particular interest because it can be administered in vivo, without the side effects, including emesis, typical of first and second generation inhibitors [71,72].

Because PDE4 represents the isoenzyme with a wider tissue distribution, most research has been focused on PDE4 inhibitors, but recently new molecules against other cAMP specific PDEs have been proposed as effective drugs in different pathological conditions. For example, a new class of PDE7 inhibitors reduces the inflammatory response and the severity of spinal cord injury [73].

## 4. Role of cAMP Signalling in Non-Hepatic Tumours

It is well known that increasing intracellular concentrations of cAMP may arrest growth, induce apoptosis and attenuate cell migration of many types of cancer cells [74,75]. A large number of papers have demonstrated a critical involvement of EPAC1 in invasion and metastasis of several cancers. EPAC1 is over-expressed in various melanoma cell lines, in which it induces cell migration through the modification of heparan sulphate chains and Ca^2+^-dependent mechanisms [76,77]. The effects of EPAC1 on cell proliferation was also established in ovarian, pancreatic and lung cancers [78], although it has been shown to be also dependent on cell type and experimental conditions. In fact, in prostate cancer cells, the specific EPAC1 activator 8-pCPT triggers the induction of B-Raf1/MEK/ERK and mTOR signalling pathways, essential for cell proliferation [79]. Conversely, in the same cell lines, the use of higher concentrations of the same EPAC1 agonist show anti-mitogenic and anti-migratory effects [80]. 

Recent studies suggest that EPAC1 can induce the migration of lung cancer cells by increasing the expression of histone deacetylase 6 (HDAC6), which in turn causes a decrease in α-tubulin acetylation and an increase in microtubule dynamics [81]. Cyclic AMP modulates the expression of HDAC8 as well. Interestingly, the use of specific shRNAs demonstrated an EPAC2 (but not EPAC1) mediated effect on HDAC8 expression in lung cancer cells [82].

The two primary cAMP effectors, EPAC and PKA, can function antagonistically, independently or synergistically to modulate cancer cell proliferation, apoptosis, and migration [83,84]. 

The PKA pathway has been demonstrated to stimulate cell growth in different cell types, while inhibiting others [85]. The different effects are probably due to the expression of two distinct isoforms of PKA (I and II) differing in their regulatory subunits. Overexpression of the PKA-I isoform, with a loss of balance with PKA-II, is considered a hallmark of most human tumours, correlating with more serious pathological features [86]. Constitutive activation of PKA-I induces immortalization in mouse embryonic fibroblasts through upregulation of D-type cyclins and reduction of autophagy [87]. On the other hand, overexpression of the regulatory subunit IIβ inhibits cell growth in human colon carcinoma [88].

As already said, the easiest way to increase the levels of cAMP is through inhibition of degradative enzymes, and of PDE4 in particular. It is not surprising that already in a study of 20 years ago, in which 60 tumour cell lines were analysed, 41 of these showed increased hydrolytic activity towards cAMP compared to the non-tumour counterpart, with a significant overexpression of PDE4 [89]. 

In a more recent study, Lin and coauthors [90] showed recurring microdeletions in the PDE4D gene without loss of mRNA expression in about 4% of solid tumours. In addition, immuno-histochemical staining revealed an overexpression of PDE4D in several different types of primary tumour samples. Of note, depletion of endogenous PDE4D with three independent shRNAs, or treating with the specific inhibitor 26B, caused apoptosis and growth inhibition in breast, lung, ovary, endometrium, gastric and melanoma cancer cells, which could be rescued by re-expression of PDE4D. The events were associated with induction of Bcl-2 and downregulation of the microphthalmia-associated transcription factor (MITF) in a lineage-dependent manner. Furthermore, ectopic expression of PDE4D2, a PDE4D short isoform, enhanced cancer cell proliferation, both in vitro and in vivo. The authors propose that PDE4D may function as a tumour-promoting factor and may represent a unique target for cancer cell therapy [90].

Interestingly, in pancreatic cancer cells resistant to most chemotherapy drugs, PDE4 inhibitors reduce cell proliferation and increase apoptosis in a caspase-dependent manner [91]. A combination of rolipram and low doses of forskolin causes growth arrest of the colon-resistant tumour cells KM12C [92]. Furthermore, the PDE4 DC-TA-46 inhibitor is effective in controlling the growth of neuroepithelioma cells [93] and other human tumours, as well as in mouse lung cancer cell lines [94,95], thus representing a promising tool in anticancer treatments. However, overall, DC-TA-46 has been little used in research and has only recently been considered in hepatocellular carcinoma studies [13]. 

Other second-generation PDE4D inhibitors have been extensively tested in human clinical trials; among them, cilomilast, already approved for the treatment of respiratory disorders, has also been tested in prostate cancer models. Cilomilast treatment strongly decreases the growth and the tumour size in in vivo and in vitro studies, reducing the expression of hedgehog activated genes [96]. 

Much attention has been paid to brain tumours since, due to the blood-brain barrier (BBB) and to their intrathecal position, they are particularly difficult to treat with traditional chemotherapy. PDE inhibitors can cross the BBB and act on the central and peripheral nervous system. In fact, many PDE inhibitors are likely candidates for the treatment of many neuro-pathologies such as depression, schizophrenia and Parkinson′s disease [66,97,98] and also as “memory and cognition enhancer” [99,100,101]. A series of experimental results show that selective inhibition of PDE4 suppresses the growth of brain tumour cells and increases the anti-tumour effects of chemotherapy and ionizing radiation therapy [102]. PDE4 is also overexpressed in tumours of the nervous system and promotes the growth of glioblastomas, medulloblastomas, ependymomas, oligodendroglioma, meningioma [103], and of the childhood cancer neuroblastoma [104]. 

In recent work on glioblastoma cancer stem-like cells (GCSCs), able to promote their own proliferation by secreting vascular endothelial growth factor A (VEGF-A) in an autocrine manner, positively regulated also by PDE4, the authors demonstrate that rolipram and bevacizumab, a VEGF-A factor blocker, act in a synergistic manner, inducing glioblastoma cell death [105]. Bevacizumab alone suppressed GCSC survival and increased apoptosis by triggering increased levels of p53, of cleaved-caspase 3 and ERK protein expression, along with a decrease in free VEGF-A protein. Co-treated cells showed a more substantial decline in free VEGF-A levels and a greater increase of p53 and cleaved-caspase 3 compared to those treated with bevacizumab alone [105]. PDE7B is another cAMP specific phosphodiesterase that is frequently upregulated in glioblastoma and able to induce the in vitro expansion of cancer stem-like cells [106]. The overexpression of PDE7B has a pro-tumorigenic effect in vivo and negatively correlates with survival of patients with astrocytoma [106]. All these observations, among others, confirm that cAMP suppression may be a critical mediator of tumorigenic mechanisms.

## 5. Role of Cyclic AMP in Hepatocellular Carcinoma

From what has been said, a certain amount of data is present in the literature indicating a clear relationship between intracellular cAMP levels and development of different types of non-hepatic cancers. In contrast, data on modulation of this important second messenger in hepatocellular carcinoma are limited.

**Figure 2:**
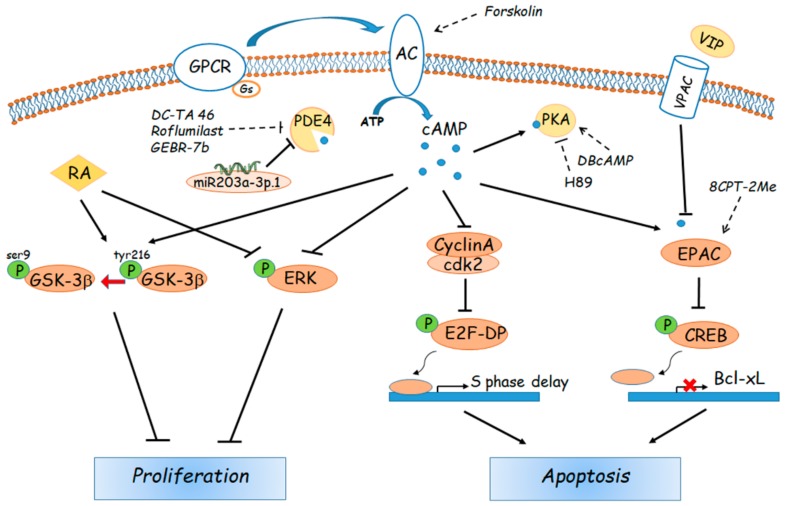
Schematic representation of the main pathways and down-stream effectors involved in cAMP signalling in HCC. GPCR, protein G coupled receptor; VPAC, vasoactive intestinal polypeptide receptor 1 or VIPR1; RA, retinoic acid; CREB, cAMP response element-binding protein; cdk2, cyclin-dependent kinase 2; GSK-3β glycogen synthase kinase 3β; ERK, extracellular-signal-regulated kinase.

Most studies used specific inhibitors of PDE or of adenylate cyclases to examine how variations of these specific enzymes and, indirectly of intracellular cAMP, affect tumour growth and metastasis; others use instead cAMP or non- hydrolysable cAMP analogues (Figure 2).

In one of the early works, the effect of cAMP modulation was studied using the cAMP analogue 8-bromo cAMP and butyryl cAMP. Treatment of the HepG2 cell line with these compounds caused a significant negative regulation of cell growth with a G1 synchronization of cells, which went through apoptotic death after prolonged drug exposure. The induction and extension of the apoptotic process was evaluated by analysing DNA condensation and fragmentation in DAPI-stained cells and by flow cytometric detection of cells with a hypodiploid DNA content [107]. Surprisingly, cAMP analogues did not induce effects on the levels of most key cell cycle proteins, such as D-type cyclins, cyclin E, CDK2, CDK4, p53 or cyclin dependent kinase inhibitors p21 or p27, but they strongly reduced cyclin A activity, protein and mRNA levels. Cyclin A is an S- and G2-M-phase regulatory protein, and its abnormal expression has been directly implicated in cellular transformation [108]. These results correlate well with findings obtained in patients with primary liver cancer, clearly showing a significant positive correlation between cyclin A mRNA tissue level and the number of hepatic tumour cells [109]. Cyclin A is rate-limiting for G1-S transition and S phase progression, but it is also required in G2/M transition. Cyclin A forms a complex with cdk2 kinase in G1 and S phases and with cdk1 kinase in G2 and early M phase [110]. Phosphorylation of the transcription factor heterodimer E2F-DP by the cyclin A-cdk2 complex results in release of E2F-DP from its DNA binding site with a consequent block of DNA synthesis. In cAMP-treated HCC cells, loss of cyclin A/cdk2 activity and of subsequent phosphorylation may induce cell death via apoptosis [107]. 

Chao and co-authors [111] performed a disease-free survival analysis, which allowed them to confirm the role of cyclin A in HCC tumours. In this paper, patients whose tumoral cells overexpressed cyclin A had a median disease-free survival of 6 months, whereas patients who lacked cyclin A overexpression exhibited a longer median disease-free survival of 29 months. Data suggest that cyclin A overexpression correlates with the degree of aggressiveness and of response to treatment. 

More recently, in our laboratory we tested the potential of the selective inhibitors of PDE4 rolipram and DC-TA-46 as molecules able to interfere with cell proliferation by increasing intracellular cAMP [13]. We firstly demonstrated that proliferating HepG2 cells have a higher total PDE activity than non-proliferating HepaRG cells. Moreover, biochemical analysis proved that PDE4 activity accounts for almost half of the total HepG2 cAMP-PDE activity and that depends mostly on the expression of three PDE4 isoforms, A, B and D. In particular, isoforms A and D were up-regulated in HepG2 cells compared with HepaRG and normal rat hepatocytes. Treatment with inhibitors caused a marked increase in intracellular cAMP levels and affected HepG2 cell growth in a dose-and time-dependent manner. In our experiments, the inhibition of cAMP-PDE with rolipram and DC-TA-46 caused a significant decrease of cyclin A expression, but also an increase of p21, p27 and p53. Both inhibitors caused apoptosis as demonstrated by an Annexin-V cytofluorimetric assay and analysis of caspase-3/7 activity. In addition, changes in the intracellular localization of cyclin D1 were also observed after treatments [13]. These results strongly suggest that the use of PDE4 inhibitors, which are characterized by elevated activity and low toxicity, may provide a challenging strategy for the treatment of HCC, in agreement with results obtained in other malignant cell types [93,112]. Rolipram has also been tested in clinical trials and in clinical development for various therapeutic indications at well tolerated doses [113,114]. Aberrant overexpression of PDE4 isoforms A and D has also been found in other hepatocarcinoma cell lines, including Huh7 and Hep3B, causing them to be proposed as prognostic elements and potential therapeutic targets for HCC [14,15]. 

A different strategy for the use of PDE inhibitors is to evaluate their synergistic effects with commonly used chemotherapeutics and/or with molecules able to enhance their effects. Ionta et al. [115] evaluated the effect of cAMP alone or combined with retinoic acid (RA) on the growth of the HCC cell line HTC. RA is an active metabolite of vitamin A that regulates important biological process such as proliferation and differentiation as well as cell death [116]. It is well known that RA affects cell proliferation of several HCC cell lines with different responsiveness depending on the cell type [117,118,119,120]. RA treatment stimulated proliferation of Hep3B and SNU449 cells but significantly inhibited cell growth in HepG2, SNU354 and HTC, confirming that the responsiveness of drugs depends on typical cancer biochemical features that can be very different even in closely related cells [115,119]. By using HTC cells, Ionta and co-authors [115] demonstrated that RA and cAMP were both effective in inhibiting the proliferation of HTC cells. However, the combined use of RA and cAMP prolonged the period of inhibition and was more effective in inducing cell differentiation, as demonstrated by the increase of E-cadherin, Cx26, Cx32 and Ser9-GSK-3β (inactive form) expression, and by the decrease of Cx43, Tyr216-GSK-3β (active form) and phosphorylated ERK. Furthermore, telomerase activity was also synergistically inhibited by the combined treatment. 

A different signalling pathway is triggered by the vasoactive intestinal peptide (VIP), a modulator of inflammatory responses, whose receptors, VPAC1 and VPAC2, are overexpressed in many cancer cells including HCC, probably due to the inflammatory state associated with the development of tumours. VIP binds to G protein-coupled receptors and leads to activation of the cAMP/PKA pathway [121]. 

In a recent paper, Hara and co-authors [122] found that VIP efficiently blocks the proliferation of the HCC cell model Huh7 through a PKA-independent mechanism. VIP reduced cAMP concentration, ser133-phosphorylation on CREB and CREB protein levels, and significantly triggered apoptosis of Huh7 by inhibiting the cAMP/CREB/Bcl-xL pathway. The effects were reversed by addition of VIP receptor antagonists and the cAMP antagonist Rp-cAMPS but also by pre-treatment with a cAMP analogue, causing the involvement of a PKA-independent signalling mechanism to be assumed. The authors suggest that the signalling pathway mediated by EPAC may be responsible for the VIP-induced apoptosis, in agreement with previous studies on human renal carcinoma cells [123].

A potent and selective anti-tumoral effect on sensitive HCC cells was demonstrated also for zardaverine, a dual-selective PDE3/4 inhibitor [124]. The effect was consequent to blockage of the cell cycle in the G0/G1 phase, to the dysregulation of important proteins, including Cdk4, Cdk6, Cdk2, Cyclin A, Cyclin E, p21 and Rb, and to the induction of apoptosis through cleavage of caspase-3, 8 and 9. In addition, the level of Rb protein, which functions in preventing the entrance of cells into S phase, was closely related to the resistance of cells to zardaverine–resistant cells had higher levels of Rb than sensitive cells, with HepG2 being among the most resistant cells. More interestingly, the antiproliferative effect was independent of the modulation of cAMP and was not inhibited by rolipram, although this inhibitor was successful in increasing the levels of intracellular cAMP in the same sensitive cell lines. A possible explanation may lie in the fact that sensitive cell lines express a different pattern of PDE4 splice variants, with low levels of isoforms accountable for cell proliferation, which remain sensitive to rolipram inhibition (e.g., PDE4A isoforms) [125]. 

Finally, recent papers suggest that the cAMP-PDE4 pathway may also be modulated by micro RNAs (miRNAs), a large class of endogenous small noncoding RNAs, 21–25 nucleotides, that regulate gene expression by binding to the 3′-untranslated region (UTR) of mRNAs, leading to degradation or translational inhibition of the corresponding mRNAs [126]. It has been reported that many miRNAs play pivotal roles in the regulation of cancer cell proliferation, differentiation, apoptosis, migration and invasion, by interfering in a variety of pathways [127,128]. MiRNAs are also active participants in HCC development and progression. MiR203a-3p.1, in particular, is up-regulated in HCC and acts as an onco-miRNA, promoting cell proliferation and metastasis, by directly targeting and repressing IL-24, a well-known anti-tumour, anti-angiogenic and anti-metastatic cytokine. MiR 203a-3p.1 and IL-24 then emerge as potential drug targets for HCC [129,130]. 

MiR203a-3p was found to be up-regulated also in colorectal cancer, where it promotes proliferation, invasion and migration by suppressing expression of PDE4D. In these tumour cells, PDE4D is thus required to maintain the cell differentiated state and prevent cancer development [131]. In contrast to these finding, Kim et al. [132], in agreement with other studies, found that aberrant PDE4D expression contributed highly to the malignant phenotype of the colorectal cancer cells DLD-1 by targeting the mTOR-Myc axis; treatment with PDE4 inhibitors suppressed mTOR/Myc signalling and tumour effects; similar positive consequences were obtained when the mTOR inhibitor rapamycin was used. This study thus confirms the theory that cancer cells depend on low levels of cAMP. Whether the aberrant PDE4 expression is controlled by miR203a-3p or other miRNAs remains to be investigated in these cells, as well in HCC cells.

In the latter, a clear analysis of the relationship among MiRNAs, IL-4 and PDE expression also remains an intriguing issue to deeply understand the complex network of factors regulating proliferation, differentiation and apoptosis in HCC cells.

## 6. Concluding Remarks

Since its discovery, the cAMP signalling pathway has emerged as an evolutionarily highly conserved mechanism, involved in regulation of numerous physiological responses within the cell. Through several different effector proteins, cAMP levels can effectively determine the functional status of cells in health and disease. By interacting with components of other signalling pathways, cAMP plays important roles also in controlling cell growth in normal and transformed cells, with tasks that depend on the cell type and specific contexts. In most tumour cells, including HCC cells, cAMP acts as a controller of cell proliferation. An inverse relationship between cAMP levels and degree of malignancy, as well as an overexpression of PDEs, have been found in these cells. This review provides experimental evidence that raising cAMP levels may help to counteract HCC development and its effects. Currently, the use of PDE inhibitors instead of adenylate cyclase activators appears to be the most rational approach, as data from the literature indicates that the inhibition of their degradation is more efficient than the stimulation of their synthesis. Various specific phosphodiesterase inhibitors can block the progression of the cell cycle in many types of cancers and appear to be promising also for the treatment of HCC. Nonetheless, more studies are necessary to find new inhibitors as well as new molecules effective in modulation of key effectors in cAMP/PKA or cAMP/EPAC signalling pathways, to be used alone or in combination with other therapeutics to improve HCC clinical outcomes.
